# Rosuvastatin induced photolocalized purpura

**DOI:** 10.1002/ccr3.6375

**Published:** 2022-09-23

**Authors:** Marwa Thabouti, Nadia Gahriani Fetoui, Linda Manaa, Jacem Rouatbi, Badreddine Sriha, Chaker Ben Salem, Mohamed Denguezli

**Affiliations:** ^1^ Dermatology Department Farhat Hached Hospital Sousse Tunisia; ^2^ Anatomopathology Department Farhat Hached Hospital Sousse Tunisia; ^3^ Pharmacology Department Farhat Hached Hospital Sousse Tunisia

**Keywords:** photolocalized purpura, photosensitivity, rosuvastatin

## Abstract

Statins are a widely used class of drug, usually safe and well‐tolerated. Their cutaneous side effects are exceedingly rare. We describe a case of photoexposed purpuric eruption mediated by rosuvastatin.

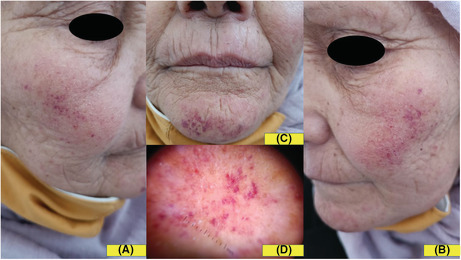

Dear Editor,

Statins are a widely used class of drug, usually safe and well‐tolerated. Their cutaneous side effects are exceedingly rare. We describe a case of photoexposed purpuric eruption mediated by rosuvastatin.

A 65‐year‐old woman presented with a 6 days history of pruritic eruption of the face. She had a clinical history of hypercholesterolemia diagnosed one month ago and treated by rosuvastatin 10 mg once a day. Fifteen days after the treatment was started, the patient reported the occurrence of a pruriginous eruption on the face. Dermatological examination showed isolated and confluent punctate lesions on an erythematous background, located on the cheeks, the tip of the nose, and the chin (Figure 1A–C). Dermoscopic examination revealed red dots and globules that confirmed the vascular nature of skin lesions (Figure 1D). Personal and family medical history was negative for photomediated diseases. Her laboratory parameters were within normal limits. Histopathological examination showed lymphocytic infiltrate around capillaries in the dermis without signs of vasculitis. There was a marked degree of extravasation of red blood cells (Figure 2A). No immunoreacting deposits were found in direct immunofluorescence studies. The diagnosis of photoexposed purpuric eruption induced by rosuvastatin was made, based on clinical, dermoscopic, and histopathological findings. Rosuvastatin was discontinued and the patient was prescribed topical betamethasone dipropionate 0.05% cream twice daily for three weeks, with photoprotection measures. The skin eruption completely resolved within 10 days (Figure 2B).

**FIGURE 1:**
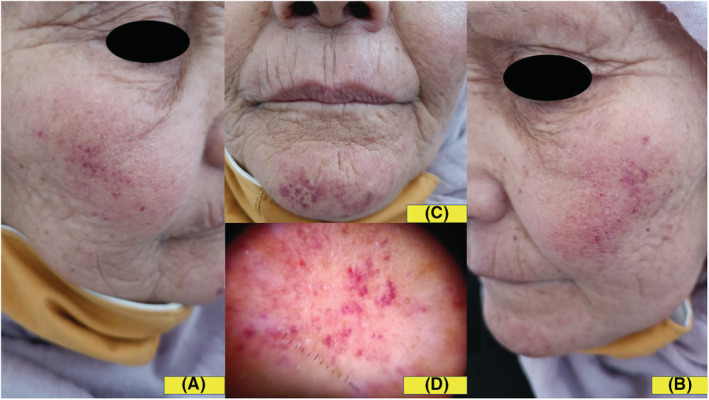
(A,B,C): Punctate lesions on an erythematous background, located on the cheeks, the tip of the nose, and the chin. (D): Red dots and globules in dermoscopic examination

**FIGURE 2:**
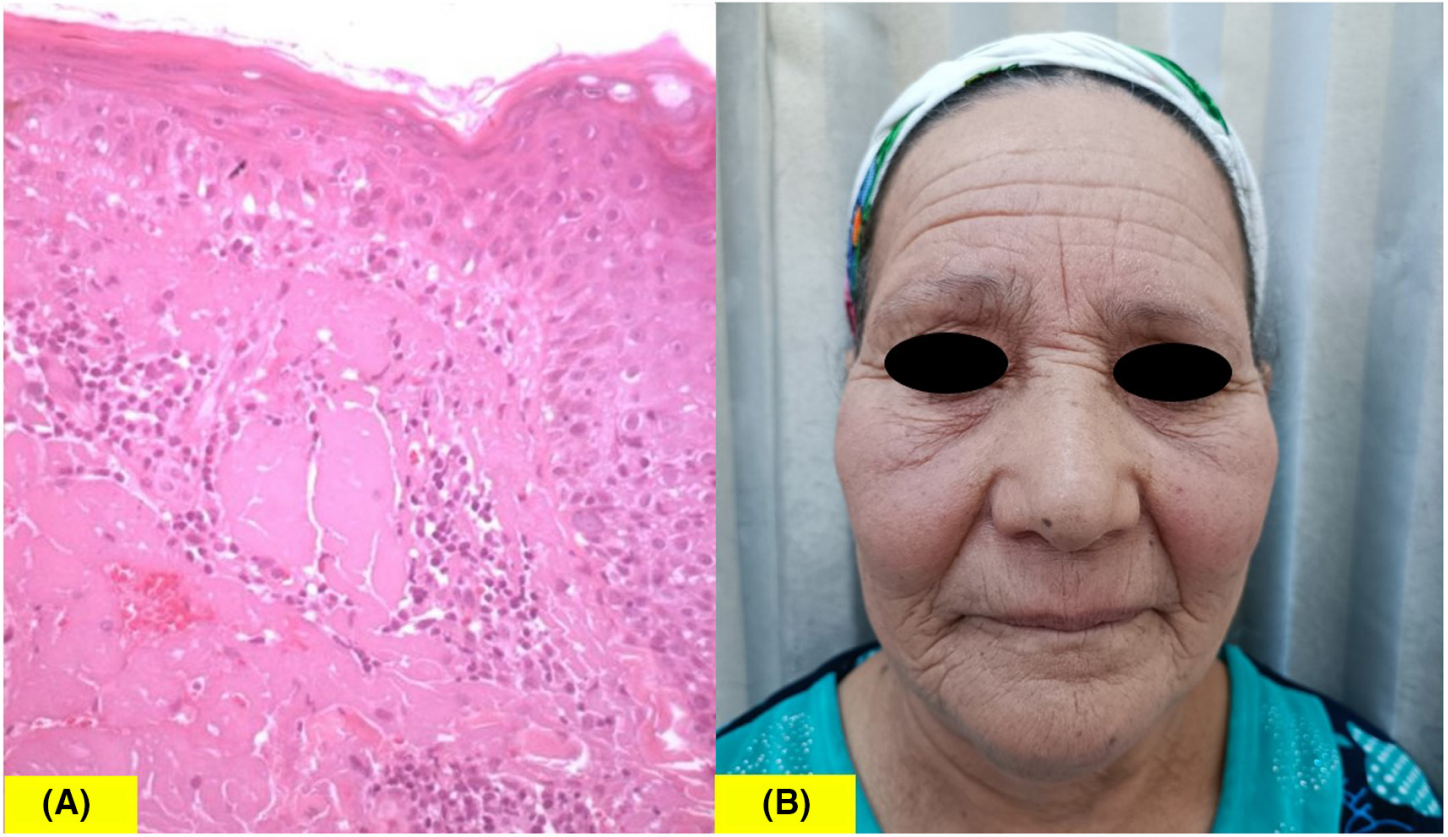
(A): HE*200: Lymphocytic infiltrate around capillaries in the dermis and extravasation of red blood cells without signs of vasculitis, (B): The skin eruption completely resolved

3‐Hydroxy‐3‐methylglutaryl coenzyme A reductase inhibitors (statins) are extensively used for the primary and secondary prevention of atherosclerotic cardiovascular events. A wide spectrum of cutaneous reactions has been described with statins, notably, acral cutaneous vesiculobullous and pustular lesions mainly with simvastatin,^1^ psoriasis‐like eruptions with pravastatin,^2^ lichenoid eruptions with atorvastatin, simvastatin, and rosuvastatin,^3^, ^4^ and other skin conditions such cutaneous lupus erythematosus, porphyria cutanea tarda, bullous dermatosis, acute generalized exanthematous pustulosis, cheilitis, and dermatomyositis‐like syndrome.^5^ Statins are less likely to induce photosensitivity. A few cases of photolocalized erythema multiform have been described with simvastatin and pravastatin; eczematous and lichenoid photosensitivity with fenofibrates and chronic actinic dermatitis has been described with simvastatin.^6^

Our patient presented a petechial purpura strictly limited to sun‐exposed areas, notably her face, without vasculitis, 15 days after starting rosuvastatin therapy. This uncommon skin reaction was only described with levofloxacin and ciprofloxacin therapy.^7^, ^8^

To the best of our knowledge, this is the first case of photolocalized purpuric eruption associated with Rosuvastatin. Its etiopathogenesis is not yet elucidated. Physicians should be aware of this skin reaction, which could be added to the spectrum of statin induced photosensitivity eruptions.

## AUTHOR CONTRIBUTION

Marwa Thabouti wrote the manuscript. Nadia Gahriani Fetoui revised the manuscript. Linda Manaa contributed to the management of the patient. Jacem Rouatbi and Badreddine Sriha contributed to the anatomopathological examination. Chaker Ben Salem contributed to the pharmacological study. Mohamed Denguezli reviewed the manuscript and gave final approval.

## CONFLICTS OF INTEREST

The authors have no conflicts of interest to declare.

## CONSENT

“Written informed consent was obtained from the patient to publish this report in accordance with the journal's patient consent policy.”

## Data Availability

none
